# Comparisons of efficacy, safety, and recurrence risk factors of paroxysmal and persistent atrial fibrillation catheter ablation using robotic magnetic navigation system

**DOI:** 10.1002/clc.23156

**Published:** 2019-03-15

**Authors:** Xiao‐Xi Zhao, Ku‐Lin Li, Ru‐Xing Wang, Jie Zheng, Xiao‐Yu Liu, Shi‐Peng Dang, Zhi‐Ming Yu, Chang‐Ying Zhang, Xiang‐Jun Yang

**Affiliations:** ^1^ Department of Cardiology The First Hospital Affiliated to Soochow University Suzhou Jiangsu China; ^2^ Department of Cardiology Wuxi People's Hospital Affiliated to Nanjing Medical University Wuxi Jiangsu China

**Keywords:** atrial fibrillation, catheter ablation, magnetic navigation system, recurrence, risk factor

## Abstract

**Background:**

No data exist on comparisons of efficacy, safety, and recurrence risk factors of paroxysmal and persistent atrial fibrillation (AF) ablation using robotic magnetic navigation system (MNS), respectively.

**Methods:**

About 151 AF patients were prospectively enrolled and divided into paroxysmal AF group (n = 102) and persistent AF group (n = 49). Circumferential pulmonary vein antrum isolation (CPVI) was performed in all patients. Linear ablation at the left atrial roof and mitral isthmus was performed in patients with persistent AF in addition to CPVI. The procedural time, X‐ray exposure time, acute and long‐term success rates of CPVI, and procedure‐related complications were analyzed. The AF recurrence rates in the two groups were compared during 1 year, and Cox regression was used to analyze the recurrence risk factors.

**Results:**

The acute success rates of CPVI in the two groups were 98.04% and 97.96%, respectively. There were no significant differences in the procedural time, X‐ray exposure time, and ablation time between the two groups (*P* > 0.05). No serious complications appeared in either group. The AF ablation success rates were 70.6% and 57.1% for the paroxysmal and persistent groups respectively at 12‐month follow‐up (*P* = 0.102). AF duration and coronary heart disease prior to ablation were associated with the higher AF recurrence in patients with persistent AF.

**Conclusion:**

Ablation using MNS is effective and safe both in patients with paroxysmal and persistent AF. AF duration and coronary heart disease prior to ablation are two independent risk factors of AF recurrence in patients with persistent AF postoperatively.

## 1 INTRODUCTION

Atrial fibrillation (AF) is the most common sustained arrhythmia, and it is associated with higher mortality and morbidity,^1^ with similar outcomes amongst current treatment strategies. Currently, catheter ablation has become one of the preferred methods for AF treatment^2^, ^3^; however, manual AF ablation has some disadvantages that include a complex procedure, long procedural times, long learning curve, risk of serious complications, and high recurrence rate. Consequently, finding new techniques for AF ablation that provide clinical benefit for the treatment of AF patients is important.

In the recent decade, ablation using the Niobe II robotic magnetic navigation system (MNS) (Stereotaxis, Inc. St. Louis, Missouri) has been one of the novel techniques used to address the challenges of AF ablation procedures.^4^, ^5^, ^6^ MNS procedures have fewer complications and reduced fluoroscopy exposure time to patients, physicians and Ep lab staff. Many studies have demonstrated that ablation using MNS is noninferior to manual ablation in efficacy and safety.^7^, ^8^ On the other hand, as we expected based on numerous prior studies, the efficacy of manual ablation was lower for persistent than for paroxysmal AF. However, there are still no data on comparisons of efficacy and safety of paroxysmal and persistent AF ablation using MNS respectively. Therefore, the aim of this study was to investigate the efficacy, safety, and recurrence risk factors of catheter ablation using MNS for paroxysmal and persistent AF, respectively.

## 2 METHODS

### 2.1 Study population

In this prospective study, a total of 151 consecutive AF patients, including 102 paroxysmal and 49 persistent AF patients, with mean age of 63.9 ± 7.8 (18‐77) years, were enrolled for AF ablation using MNS from October 2012 to February 2017. These patients were all first time ablation procedures, and no redo patients were included.

### 2.2 Criteria of inclusion and exclusion

Inclusion criteria were patients with drug‐refractory symptomatic AF, more than one risk factor of thrombosis, and no thrombus found by transoesophageal echocardiography examination in left atrium prior to ablation. Exclusion criteria were patients with ages >80 or < 18 years, severely decompensated heart failure (ejection fraction (EF) ≤40%), left atrium diameter ≥ 55 mm, contraindications to anticoagulation, severe hepatic, renal or lung dysfunction, hyperthyroidism, and thrombus in the left atrium illustrated on either echocardiography or computed tomography (CT) scan. The potential risks of the procedure were explained, and written informed consent was obtained from all patients. Owing to good efficacy and better safety, AF ablation was performed in all cases using MNS in our center. This study was reviewed and approved by the Institutional Review Board of Wuxi People's Hospital affiliated to Nanjing Medical University, China.

### 2.3 Definitions of paroxysmal AF and persistent AF

In this study, AF was categorized as paroxysmal and persistent. Paroxysmal AF was defined as self‐terminating within 7 days or terminated with pharmacological or electrical cardioversion; persistent AF was defined as lasting >7 days, requiring cardioversion or other intervention or failed cardioversion, or cardioversion was no longer attempted.

### 2.4 Electrophysiological study and catheter ablation

The MNS Niobe II system (Stereotaxis Inc.) is composed of two focused‐field permanent magnets, irrigated magnetic catheter and Cardiodrive catheter advancing system (QuikCAS; Stereotaxis, Inc.). The two large magnets with 0.08 to 0.1 T magnetic field are located on either side of the patients' body. The magnetic catheter can be actively deflected by changing the magnetic field orientations. The third‐generation irrigated magnet catheter (Navistar Thermocool RMT; Biosense Webster, Inc. Diamond Bar, California) can be steered leftwards or rightwards following the direction of the vectors by magnet field‐controlled system, and can be advanced or retracted with minimal 1 mm step by mechanical device. Three‐dimensional electroanatomic mapping along side an X‐ray image (CARTO RMT; Biosense Webster Inc.) can be displayed on MNS monitor. Ablation was performed remotely in the control room, away from radiation exposure, by clicking the computer mouse and keyboard of MNS.

**Figure 1:**
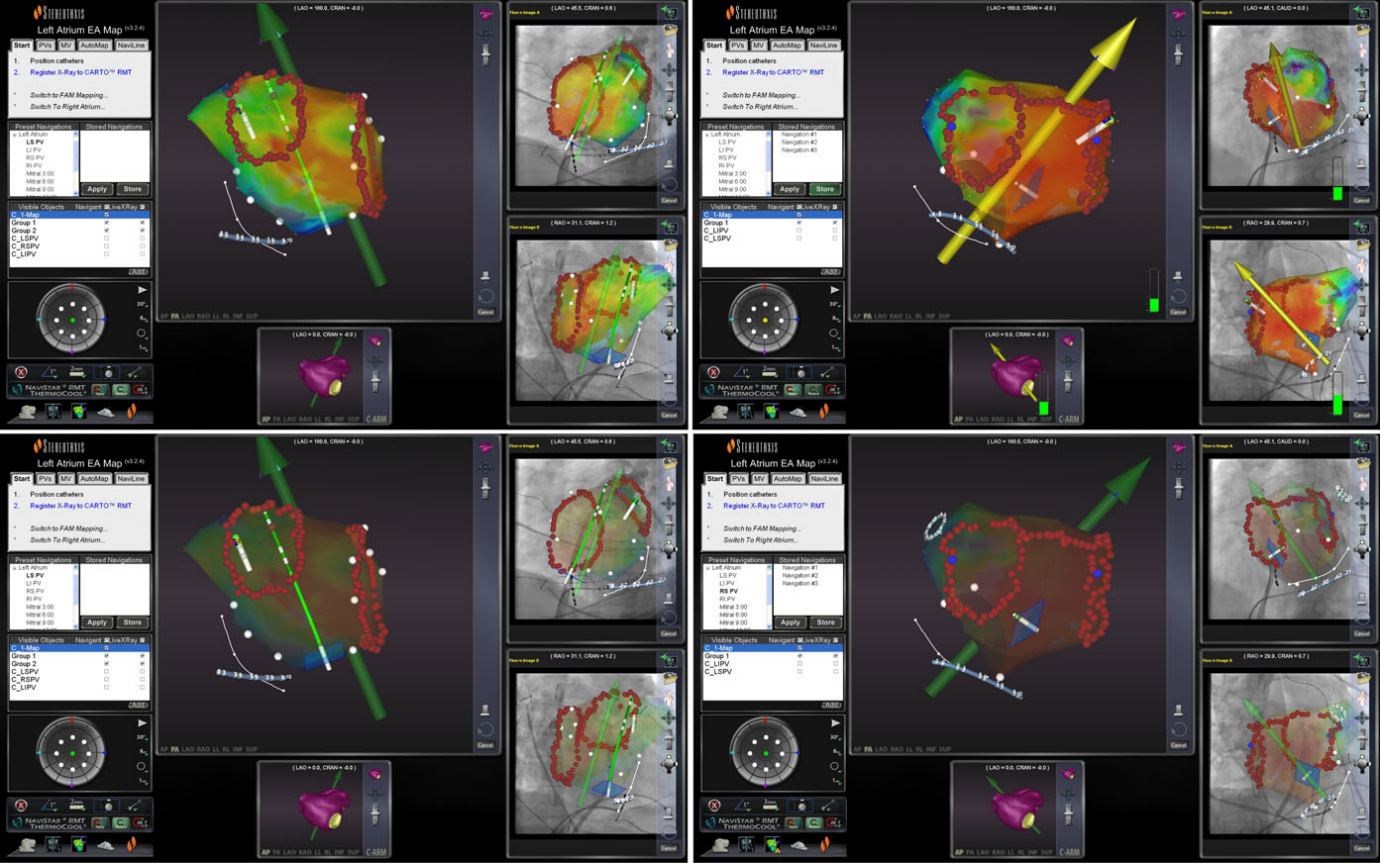
Catheter ablation of paroxysmal (left) and persistent (right) atrial fibrillation using magnetic navigation system. Left panel is paroxysmal atrial fibrillation ablation using magnetic navigation system, and the ablation protocol is only circumferential pulmonary vein antrum isolation. Right panel is persistent atrial fibrillation ablation using magnetic navigation system, and the ablation protocol is the linear ablation of mitral isthmus and left atrial roof in addition to circumferential pulmonary vein antrum isolation. The upper panel and the lower panel in left and right are the pictures at the same time, but the upper panel can simultaneously show three‐dimensional model of left atrium and ablation lines on fluoroscopy monitor, and the lower panel only shows ablation lines on fluoroscopy monitor

The decapolar catheter (Inquiry, St. Jude Medical, Inc. St. Paul, Minnesota) was routinely placed in coronary sinus via the right femoral vein. Transseptal puncture was done under the fluoroscopic guidance, and the angiographies of left and right pulmonary veins were performed after atrial septal puncture. The MNS catheter, used both for mapping and ablation, was moved to left atrium through the sheaths (Swartz SR0, St. Jude Medical Inc.). Using the preset navigation feature, the catheter can virtually move automatically to the target pulmonary veins after clicking the short‐cut menu. Heparin was given to maintain ACT of 250 to 300 seconds during the whole ablation procedure. Circumferential pulmonary vein antrum isolation (CPVI) only was performed in patients with paroxysmal AF. Linear ablation at left atrial roof and mitral isthmus was done in patients with persistent AF in addition to CPVI (Figure 1). Electrical cardioversion was carried out with failure to maintain sinus rhythm after ablation. The duration of radiofrequency ablation on each point was set at 30 to 60 seconds, or the potential amplitude declined by 80%. The ablation was performed with the temperature setting at 43°C, power of 30 to 35 W for the anterior wall and 25 to 30 W for the posterior wall, with a flow rate of 17 mL/min during ablation and 2 mL/min during ablation free interval. Finally, CPVI was confirmed by Lasso catheter at each pulmonary antrum. Procedure‐related parameters such as acute success rate of CPVI, procedure time, mapping and CPVI isolation time, radiofrequency ablation time, exposure to X‐ray time, and ablation complications were recorded.

### 2.5 Follow‐ups

Follow‐ups were performed for all patients in outpatient clinic or by telephone interviews at 1, 3, 6, 9, and 12 months post procedure. Oral anti‐coagulation therapy with a target international normalized ratio between 1.8 and 2.5 and anti‐arrhythmic drugs were administered for 2 to 3 months after the procedure. In addition to the clinic follow‐up and telephone interview, 24‐hour Holter monitoring was routinely performed every 2 months post‐ablation in order to obtain the patient clinical manifestations and the occurrence of arrhythmias. The efficacy endpoints of AF ablation using MNS evaluated in this study were acute success rate of CPVI for the paroxysmal group, and CPVI plus lines for the persistent group, and the AF recurrence at 1, 3, 6, 9, and 12 months postoperatively. AF recurrence was defined as a documented episode of AF or atrial tachycardias lasting more than 30 seconds within 1 year from ablation date. The safety of AF ablation using MNS was evaluated by assessing the rate of procedure‐related complications perioperatively and the time of X‐ray exposure.

### 2.6 Statistical analysis

Continuous variables were expressed as mean values ± SD (^−^x ± s) or as median with inter‐quartile range. Categorical variables were expressed as ratios and percentages. SPSS version 22.0 (SPSS Inc, Chicago, Illinois) was used for statistical analysis. Normally distributed data were compared using the independent Student's *t* test. Non‐normally distributed data between two groups were compared using the Mann‐Whitney *U* test. The χ^2^ test or the Fisher exact test was used to measure the association for categorical variables. The Pearson or the Spearman correlation analysis was used to identify the related risk factors of AF recurrence after ablation. A Kaplan‐Meier analysis was used to compare the probability of AF recurrence in two groups. Univariate analysis and multivariate analysis were performed using Cox‐regression test to identify the risk factors of AF recurrence after ablation. A *P* value <0.05 was considered statistically significant.

## 3 RESULTS

### 3.1 Patient characteristics

The clinical baseline characteristics of patients in the two groups were nearly identical. There were no statistically significant differences between groups in terms of sex, age, weight, AF duration, heart function class, left ventricular ejection fraction, number of anti‐arrhythmic drugs, underlying diseases and lab results, but significant differences in diameters and volumes of left atrium were noted. (Table 1).

**Table 1:** Baseline characteristics of patients prior to ablation

Variable	All patients (n = 151)	Paroxysmal AF (n = 102)	Persistent AF (n = 49)	*P* value
Sex (M/F)	93/58	59/43	34/15	0.172
Age (years)	58.62 ± 10.32	59.25 ± 9.86	57.33 ± 11.21	0.290
Weight (kg)	68.57 ± 10.10	68.77 ± 11.26	70.68 ± 11.06	0.403
AF duration (month)	36	36	24	0.174
LAD (mm)	39.01 ± 5.12	37.57 ± 4.36	42 ± 5.34	0.000
LAV (mL)	105.41 ± 36.66	93.82 ± 26.84	129.54 ± 42.48	0.000
EF (%)	63.48 ± 4.45	63.92 ± 4.26	62.55 ± 4.73	0.076
Heart Function Class (NYHA)	1.25 ± 0.47	1.21 ± 0.41	1.34 ± 0.57	0.086
No. of antiarrhythmic drugs (n)	1.81 ± 0.44	1.79 ± 0.45	1.84 ± 0.43	0.573
Underlying diseases (n)				
HTN	73	49	24	0.914
CAD	23	13	10	0.220
DM	3	3	0	0.551
Hyperthyroidism	2	2	0	1.000
ACI	2	0	2	0.104
Others	2	1	1	0.545
Absence of underlying diseases (n)	46	34	12	0.269
Lab examinations				
BUN (mmol/L)	4.81 ± 1.48	4.75 ± 1.56	4.94 ± 1.32	0.450
Cr (mmol/L)	78.15 ± 16.83	76.65 ± 16.08	81.42 ± 18.11	1.110
FBG (mmol/L)	5.10 ± 1.37	4.97 ± 0.81	5.37 ± 2.12	0.100
TC (mmol/L)	4.15 ± 0.82	4.15 ± 0.79	4.15 ± 0.87	0.950
LDL (mmol/L)	2.24 ± 0.74	2.21 ± 0.75	2.29 ± 0.73	0.560
TG (mmol/L)	2.10 ± 1.14	1.90 ± 1.47	2.08 ± 1.32	0.288

Abbreviations: LAD, left atrial diameter; LAV, left atrial volume; HTN, hypertension; CAD, coronary artery disease; DM, diabetes mellitus; ACI, acute cerebral infarction; BUN, blood urea nitrogen; Cr, creatinine; FBG, fasting blood glucose; TC, total cholesterol; LDL, low‐density lipoprotein; TG, triglyceride.

### 3.2 Procedural parameters and acute success rates of CPVI

There were no remarkable differences of procedure duration, time from left atrium electroanatomic mapping to pulmonary vein isolation, time of radiofrequency catheter ablation, fluoroscopy exposure time, and the incidence of complications during the procedures between the two groups. The acute success rates of CPVI in the paroxysmal and persistent groups were 98.04% and 97.96% (*P* = 0.974), respectively (Table 2).

**Table 2:** Procedure parameters and follow‐up in two groups

Variable	Paroxysmal AF (n = 102)	Persistent AF (n = 49)	*P* value
Procedure time (min)	146.73 ± 23.05	152.45 ± 28.78	0.230
Mapping and CPVI time (min)	101.86 ± 21.17	110.31 ± 28.24	1.857
Ablation time (min)	38.75 ± 8.37	39.63 ± 7.54	0.532
X‐ray exposure time (min)	9.70 ± 5.90	10.15 ± 5.01	0.640
Sheath (n)			
SR0	99	49	0.551
SR1	3	0	0.551
Dosage of heparin (u)	5619.09 ± 1500.87	5864.58 ± 1511.44	0.353
Cases of complications (n)			
Sudden death	0	0	—
Cardiac perforation	0	0	—
Esophageal fistula	0	0	—
PVS	0	0	—
Thrombus	0	0	—
Hemothorax	2	0	1.000
Hematoma	0	1	0.327
Hemopneumothorax	0	0	—
Acute success rates of CPVI (%)	98.04	97.96	0.974
Percents of free from AF (%)			
1 mo	88.20	81.60	0.272
3 mo	83.30	75.50	0.253
6 mo	80.40	67.30	0.078
9 mo	72.50	61.20	0.159
12 mo	70.60	57.10	0.102

Abbreviations: AF, atrial fibrillation; CPVI, pulmonary vein isolation; PVS, pulmonary vein stenosis.

### 3.3 Recurrence rates of AF

**Figure 2:**
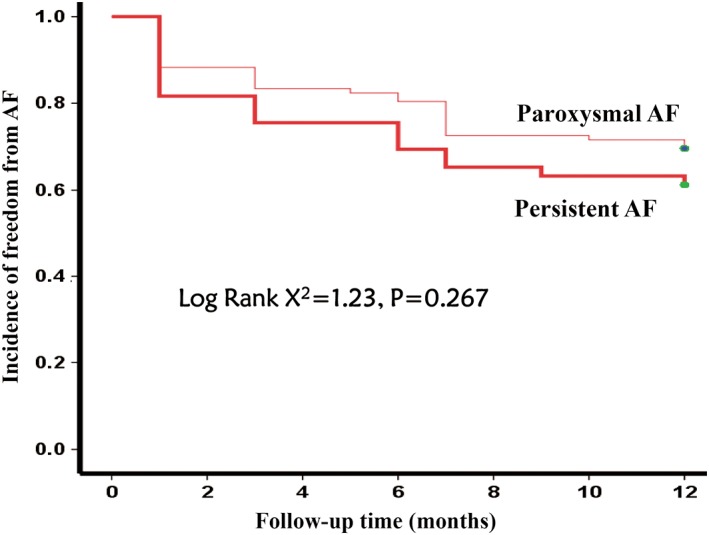
Kaplan‐Meier curve of atrial fibrillation recurrence after ablation using magnetic navigation system. A Kaplan‐Meier analysis was used to compare the probability of atrial fibrillation recurrence between the two groups. The Kaplan‐Meier analysis showed that the incidences of freedom from atrial fibrillation were 70.6% and 57.1% in patients with paroxysmal atrial fibrillation and paroxysmal atrial fibrillation respectively after 12‐month follow‐up

The recurrence rates of paroxysmal AF were 11.8%, 16.7%, 19.6%, 27.5%, and 29.4% and those of persistent AF were 18.4%, 20.5%, 32.7%, 38.8%, and 42.9% during the follow‐ups of 1, 3, 6, 9, and 12 months after ablation. There were no statistical differences in AF recurrence rates between the two groups though AF recurrence rates in patients with persistent AF tended to be higher than those in patients with paroxysmal AF during each follow up time period (Table 2). The Kaplan‐Meier analysis showed that the incidences of freedom from AF were 70.6% and 57.1% in patients with paroxysmal AF and persistent AF respectively at 12‐month follow‐up, and there were no statistical significance (Log rank X^2^ = 1.23, *P* = 0.267) (Figure 2).

### 3.4 Predictors of AF recurrence

Cox regression of univariate analysis demonstrated that the recurrence of paroxysmal AF was not associated with sex, age, weight, duration of AF, diameter of left atrium, heart function class, left ventricular ejection fraction, and some common underlying diseases (*r* < 0.3, *P* > 0.05). However, Cox regression of univariate and multivariate analysis showed that the duration of AF and coronary heart disease prior to ablation were two independent risk factors of predicting the recurrence of persistent AF postoperatively (Table 3).

**Table 3:** Risk factors of atrial fibrillation recurrence after ablation guided by magnetic navigation system

Variable	Paroxysmal AF		Persistent AF					
	Univariate analysis		Univariate analysis		Multivariate analysis			
	Score	*P* value	Score	*P* value	Wald	Risk ratio	95% CI	*P* value
Sex	2.120	0.145	2.968	0.085				
Age	0.979	0.322	0.115	0.735				
Weight	2.798	0.094	0.060	0.806				
AF duration	0.000	0.989	6.142	0.013	4.458	1.938	1.049‐3.583	0.035
LAD	0.284	0.594	2.123	0.145				
Heart functional class	0.006	0.939	4.670	0.031				
EF	0.103	0.749	4.071	0.044				
Underlying diseases								
HTN	1.109	0.292	0.471	0.492				
CAD	0.027	0.870	6.463	0.011	4.323	2.641	1.057‐6.599	0.038
DM	0.231	0.631	3.704	0.054				
Hyperthyroidism	0.028	0.867	0.474	0.491				
ACI	0.103	0.749	0.474	0.491				

Abbreviations: LAD, left atrial diameter; HTN, hypertension; CAD, coronary artery disease; DM, diabetes mellitus; ACI, acute cerebral infarction.

### 3.5 Procedural complications

No serious complications such as sudden death, cardiac perforation, cardiac tamponade, pulmonary vein stenosis, and esophageal fistula appeared in two groups. Hemothorax occurred in two patients with paroxysmal AF and groin hematoma occurred in one patient with persistent AF.

## 4 DISCUSSION

### 4.1 Main findings

AF ablation using MNS is performed routinely; however, no study has focused on efficacy, safety, and recurrence risk factors of paroxysmal and persistent AF ablation using MNS. This study is the first, to the best of our knowledge, to investigate and compare the efficacy, safety and recurrence risk factors of catheter ablation using MNS for paroxysmal and persistent AF. The main findings of this study were as follows: (a) AF ablation using MNS was effective and safe both in patients with paroxysmal AF and patients with persistent AF; (b) AF duration and coronary heart disease prior to ablation were two independent risk factors of AF recurrence in patients with persistent AF postoperatively.

### 4.2 Development of AF ablation guided by MNS

It has been proven that catheter ablation is one of the effective treatments for AF patients. However, the conventional manual ablation technique for AF ablation has potential disadvantages of complex procedures, prolonged radiation exposure to patients and operators, increased risk of complications, and lengthened learning curve and so on. Therefore, advancements to remedy these issues will have great clinical implications especially those that simplify the ablation procedure, minimize the risk of complications, reduce the fluoroscopic exposure time, and decrease the learning curve.

In the last decade, it has demonstrated that AF ablation using MNS may be one of the promising ablation techniques that may address issues surrounding conventional manual ablation techniques.^9^, ^10^ In 2006, Pappone et al^11^ first reported the MNS‐guided AF ablation. In 2007, Di Biase et al^12^ conducted a further study on feasibility and effectiveness of AF ablation using MNS and this study found that it was difficult to achieve CPVI in most of cases with non‐irrigated catheter ablation due to the formation of the char in heart. However, with the application of saline‐irrigated magnetic catheters in 2008, AF ablation using MNS came into clinical practice again.^13^, ^14^ In the last decade, many clinical trials have confirmed that there are the same acute and long‐term success rates of AF ablation, less procedural complications, and better safety in MNS‐guided AF ablation compared with the conventional manual ablation.^13^, ^14^, ^15^ Currently, MNS‐guided AF ablation is widely used in many developed countries.^16^ However, there is still no comparative study on paroxysmal and persistent AF ablation using MNS. Therefore, this study is mainly focused on efficacy, safety, and risk factors of AF recurrence for paroxysmal and persistent AF after ablation guided by MNS.

### 4.3 Efficacy of AF ablation guided by MNS

Despite different ablation protocols for patients with paroxysmal and persistent AF, there were no significant statistical differences in acute success rates of CPVI, mapping time, ablation duration, total procedure time, X‐ray exposure, and incidence of procedure‐related complications or recurrence rates in follow‐up. In our study, the recurrence rates were statistically the same between the two groups, however, trended higher in the persistent AF cohort. These results suggested that efficacy and safety were the same for paroxysmal and persistent AF ablation using MNS. The reason may be related to the following factors: (a) The MNS ablation catheter has better stability and remains in constant contact with heart tissue regardless of heart rhythm / rate and respiration, which makes a higher efficacy of ablation lesions.^17^ (b) Irrigated magnetic catheters can reach the most difficult anatomic regions of left atrium due to its tip flexibility, which improves the success rate of CPVI. In addition, computer controlled catheter advancement system allows the operators to control the catheters even with 1 mm forward or backward step, and at 1° angle to leftward or rightward, which increases the precision when ablation in the target sites. (c) These advantages may be more prominent during persistent AF ablation using MNS. In general, the sizes of left atrium in persistent AF are usually larger than those in paroxysmal AF, and it is difficult for maneuver manual catheters during the ablation procedure. However, in contrast to manual control ablation, magnetic ablation catheters in large left atrium target areas are easy to reach allowing full contact with the left or right pulmonary vein antrum, which improves the efficacy of persistent AF ablation.^18^

Owing to better stability and maneuverability of magnetic ablation catheters, many single‐center studies and meta analyses have illustrated that the acute success rates of CPVI in MNS‐guided catheter ablation is as good as manual navigation ablation,^14^, ^16^, ^19^, ^20^ and the long‐term success rate may be better during the follow‐up.^21^ Weiss JP et al^22^ performed a comparison of remote magnetic irrigated tip ablation vs manual catheter irrigated tip catheter ablation with and without force sensing feedback and found MNS resulted in outcomes similar to manual navigation. The addition of contact force sensing catheters did not improve relative procedural outcome in comparison to MNS‐guided AF ablation.

### 4.4 Safety of AF ablation using MNS

Studies have demonstrated that the serious complications such as cardiac perforation, pulmonary vein stenosis, and left atrial esophageal fistula can be significantly decreased when AF ablation is performed using MNS,^23^, ^24^ and furthermore the incidence of complications in MNS‐guided ablation was much lower, compared with conventional manual ablation.^25^, ^26^

There is significant decrease in the risk of cardiac perforation and cardiac tamponade in MNS‐guided AF ablation due to its special soft catheter. This is achieved by stable catheter‐to‐tissue contact even on the setting of constant changes of patients' heart rate and breath. In contrast, it is difficult for the operators to precisely control the contact force to the catheter lesion during manual ablation.^27^ Shurrab et al^28^ compared the safety of MNS ablation catheter and SmartTouch ablation catheter (Biosense Webster Inc.). The study showed that the incidence of complications with MNS ablation catheter was only 0.03%, while it was as high as 2.3% to 4% with SmartTouch ablation catheter. In our study, no serious complications occurred both in patients with paroxysmal AF and patients with persistent AF.

### 4.5 Risk factors of AF recurrence after ablation using MNS

AF recurrence rates after single catheter ablation may range from 30% to 50%, often requiring repeated ablation and leading to increased treatment costs. Therefore, the accurate prediction of negative events in patients undergoing AF ablation has important clinical significance. 29

There are few data on the recurrence risk factors of paroxysmal and persistent AF ablation using MNS. Sohns et al^30^ reported that pulmonary vein anatomy assessed by multidetector CT is a good predictor of AF recurrence after PVA using MNS. To explore the risk factors of AF recurrence after ablation using MNS in patients with paroxysmal and persistent AF, univariate analysis and multivariate analysis were performed using Cox‐regression test to identify the risk factors of AF recurrence after ablation. The results showed that the recurrence of paroxysmal AF following ablation using MNS was not correlated with sex, age, weight, AF duration, inner diameter of left atrium, heart function class, ejection fraction, and some common underlying diseases; therefore, the risk factors for the recurrence of paroxysmal AF still need to be further studied. Nevertheless, we found that AF duration and coronary heart disease were two independent risk factors for the recurrence of persistent AF, which means atrial fibrosis due to long‐term AF duration and myocardial ischemia may the key causes of higher recurrence in patients with persistent AF after ablation using MNS.

Recently, more attention has been paid on predictors of improvement in symptoms and health‐related quality of life after AF ablation in addition to predictors of AF recurrence.^31^ If predictors of AF recurrence are combined with predictors of improvement in symptoms and health‐related quality of life, they may better evaluate the effects of AF ablation using MNS for paroxysmal AF and persistent AF, respectively.

### 4.6 Study limitations

There are at least two limitations in this study. First, the study is a single‐center study, and the patients enrolled in this study are most of those with almost normal heart function. Second, the number of enrolled cases is not large, especially for persistent AF, only 49 cases. A multiple center study is needed to further explore efficacy, safety, and recurrence risk factors of catheter ablation using MNS for paroxysmal AF and persistent AF respectively in future.

## 5 CONCLUSIONS

Ablation using MNS is effective and safe both in patients with paroxysmal and persistent AF. AF duration and coronary heart disease prior to ablation are two independent risk factors of AF recurrence in patients with persistent AF postoperatively.

## CONFLICTS OF INTEREST

The authors have no conflicts of interest to report.
